# Antioxidants and Fertility in Women with Ovarian Aging: A Systematic Review and Meta-Analysis

**DOI:** 10.1016/j.advnut.2024.100273

**Published:** 2024-07-15

**Authors:** Yujie Shang, Nannan Song, Ruohan He, Minghua Wu

**Affiliations:** 1 School of Chinese Medicine, Hubei University of Chinese Medicine, Wuhan, China; 2 Hubei Shizhen Laboratory, Wuhan, China; 3 School of Basic Medical Sciences, Central South University, Changsha, China; 4 Key Laboratory of Carcinogenesis and Cancer Invasion of the Chinese Ministry of Education, Cancer Research Institute, Central South University, Changsha, China; 5 Liyang Branch of Jiangsu Provincial Hospital of Chinese Medicine, Changzhou, China; 6 Maternal and Child Hospital of Hubei Province, Tongji Medical College, Huazhong University of Science and Technology, Wuhan, China; 7 NHC Key Laboratory of Carcinogenesis, Xiangya Hospital, Central South University, Changsha, China

**Keywords:** antioxidants, ovarian aging, CoQ10, clinical pregnancy, oocyte, high-quality embryo

## Abstract

Ovarian aging is a major factor for female subfertility. Multiple antioxidants have been applied in different clinical scenarios, but their effects on fertility in women with ovarian aging are still unclear. To address this, a meta-analysis was performed to evaluate the effectiveness and safety of antioxidants on fertility in women with ovarian aging. A total of 20 randomized clinical trials with 2617 participants were included. The results showed that use of antioxidants not only significantly increased the number of retrieved oocytes and high-quality embryo rates but also reduced the dose of gonadotropin, contributing to higher clinical pregnancy rates. According to the subgroup analysis of different dose settings, better effects were more pronounced with lower doses; in terms of antioxidant types, coenzyme Q10 (CoQ10) tended to be more effective than melatonin, myo-inositol, and vitamins. When compared with placebo or no treatment, CoQ10 showed more advantages, whereas small improvements were observed with other drugs. In addition, based on subgroup analysis of CoQ10, the optimal treatment regimen of CoQ10 for improving pregnancy rate was 30 mg/d for 3 mo before the controlled ovarian stimulation cycle, and women with diminished ovarian reserve clearly benefited from CoQ10 treatment, especially those aged <35 y. Our study suggests that antioxidant consumption is an effective and safe complementary therapy for women with ovarian aging. Appropriate antioxidant treatment should be offered at a low dose according to the patient’s age and ovarian reserve.

This study was registered at PROSPERO as CRD42022359529.

**Statement of Significance**

This meta-analysis is the most comprehensive evaluation to date suggesting antioxidant treatment as an effective and safe complementary strategy for women with ovarian aging. Our subgroup analyses emphasize that coenzyme Q10 is a promising choice to rescue the decline in fertility caused by ovarian aging, and the optimal treatment regimen is 30 mg/d for 3 mo before ovarian stimulation. Women with diminished ovarian reserve clearly benefit from antioxidant treatment, especially those aged <35 y.

## Introduction

At present, the total fertility rate is far below the replacement level, which accelerates population aging worldwide and adversely affects public finances and social progress [1,2]. Worse still, delayed childbearing further increases the prevalence of infertility, thus exacerbating the imbalanced demographic transition [1]. Much of the problem mentioned above has mainly been attributed to a series of intractable problems caused by ovarian aging [3,4]. Ovarian aging, predominantly characterized by a progressive decline in the quantity and quality of oocytes [5], clinically manifests as diminished ovarian reserve until the loss of fertility, accompanied by endocrine dysfunction and menstrual cycle abnormalities. Due to high variability among women, ovarian aging is a complex process. Age-related ovarian aging is a natural and inevitable physiologic phenomenon. Unfortunately, numerous women suffer from ovarian aging much earlier, which is known as premature ovarian insufficiency, a state whereby the end of reproductive lifespan occurs before they are 40 y old due to a premature and irreversible loss of ovarian follicles. Considering the impaired fertility as well as the increased risks of spontaneous abortion, pregnancy-related complications, and offspring birth defects, ovarian aging is a major threat to reproductive health, leading to deleterious consequences for human well-being [6,7].

Assisted reproductive technology has been applied to alleviate infertility problems for decades. However, it does have limitations, as it circumvents, instead of directly targeting, the root cause of fertility decline—ovarian aging, such as age-related oocyte defects, which has become the most common factor for in vitro fertilization (IVF) failure. In addition, although adjunct strategies such as oocyte donation and oocyte cryopreservation have matured, the overall impact is disappointing, and they are not accessible to a considerable proportion of women for economic, ethical, and cultural reasons. Hence, evidence-based therapies that effectively and safely improve ovarian aging are needed.

The molecular basis for the deterioration of ovarian aging is multifactorial and not fully understood. The free radical theory is the classical aging theory that attributes aging phenomena to accumulated cellular oxidative stress. Reactive oxygen species, mainly produced in mitochondria, are critical for regulating various ovarian physiologic activities. The abnormal accumulation of reactive oxygen species leads to unrepaired or incorrectly repaired double-strand breaks and cellular senescence, resulting in ovarian oxidative stress and changes in the ovarian microenvironment, which cause further damage to oocyte quality and quantity [8,9]. Currently, antioxidants have been widely used in gynecologic clinical scenarios as potential therapeutic options to delay aging and improve reproductive outcomes [10]. A Cochrane review conducted in the subfertility population reported that antioxidants may improve the clinical pregnancy rate but have unclear effects on the live birth rate. Evidence based on previous studies is not convincing and instructive because they mainly focus on the general subfertility population, with the characteristics of different antioxidants and causes of infertility largely overlooked, and there is little consensus on treatment protocols, let alone the selection of an appropriate ovarian aging population that would clearly benefit from antioxidant treatment. To date, to our knowledge, no meta-analysis has evaluated the effectiveness of antioxidants in ovarian aging, and there is little consensus on treatment protocols, let alone the selection of an appropriate ovarian aging population that would clearly benefit from antioxidant treatment. All of this hinders the application of antioxidants in ovarian aging management. Therefore, there is an urgent need to determine the optimal antioxidant and administration protocols for the appropriate ovarian aging population.

Given the vital role of ovarian aging in the pathogenesis of infertility and that women with ovarian aging are the most challenging infertility group in the clinic, this systematic review and meta-analysis of randomized clinical trials (RCTs) was designed to evaluate the effectiveness and safety of antioxidants on reproductive outcomes in women with ovarian aging during IVF and further determine the optimal administration protocol, thereby providing evidence-based clinical practice recommendations to prolong reproductive lifespan and prevent ovarian aging.

## Methods

This study was conducted in accordance with the PRISMA guidelines [11] and has been registered at PROSPERO under the number CRD42022359529.

### Search strategy

Databases such as the Cochrane Central Register of Controlled Trials, PubMed, Embase, and ClinicalTrials.gov were searched from inception until 12 September, 2023. We also manually checked the references of identified studies, conference proceedings, or websites on the clinical trial registry to obtain additional potentially relevant data. There were no language or publication date restrictions. The details of the search strategy in PubMed are shown in Supplementary Table S1.

### Study selection

Studies were included if they met the following inclusion criteria: *1*) study design was a parallel-controlled RCT; *2*) evaluating the effects of antioxidants in the ovarian aging population, including women with advanced age (>35 y) and those with diminished ovarian reserve (defined as decline in oocyte quantity and quality) or premature ovarian insufficiency; *3*) RCTs with antioxidant supplementation in the experimental group compared with placebo or no treatment (with or without a cointervention). The exclusion criteria were as follows: *1*) quasi-randomized trials, cohort or case‒control studies, reviews, meta-analyses, case reports, animal or cell experiments; *2*) studies that enrolled women with any severe gynecologic diseases (e.g., abnormalities in uterine anatomy, uterine malformations, intrauterine adhesions, or endometriosis), any severe cardiovascular or cerebrovascular diseases, or psychiatric or neurologic problems; *3*) studies comparing antioxidants alone with fertility drugs; and *4*) studies that supplied insufficient statistical data on the outcomes of interest.

The primary outcomes were live birth rate (defined as the delivery of a live fetus by women randomly assigned to a given group) and clinical pregnancy rate (defined as the presence of an intrauterine gestational sac with a fetal heartbeat in the women randomly assigned to a given group). The secondary outcomes consisted of miscarriage rate (defined as pregnancy loss before 20 wk of gestation), the quality of oocytes and embryos (number of retrieved oocytes, metaphase II [MII] oocytes, and high-quality embryos) [12], and the dose of gonadotropin.

The titles and abstracts of all potential studies were scanned independently by 2 reviewers (YS and RH) to eliminate duplicated and ineligible studies. If there was insufficient information to make a decision, we sought further details from the original authors. Any discrepancies were resolved by discussion or consensus with the corresponding author.

### Data extraction

Three reviewers (YS, RH, and NS) independently performed the data extraction. Data were double-checked to minimize potential errors, and disagreements were resolved through discussion with the corresponding author. The following information was extracted: study characteristics (first author, year of publication, and location), participant characteristics (sample size, age), controlled ovarian stimulation strategies, antioxidant treatment protocols (dose, frequency, and duration), and data on the targeted outcome measures.

### Risk of bias and quality assessment

Three reviewers (YS, RH, and MW) assessed the methodological quality of eligible trials using the Cochrane Collaboration’s tool. Studies were evaluated as having a low, unclear, or high risk of bias based on the following domains: selection bias, performance bias, detection bias, attrition bias, reporting bias, and other bias. The quality of evidence was graded in light of study design, study quality, inconsistency, indirectness, and imprecision by the Grading of Recommendations Assessment, Development and Evaluation approach [13].

### Data analysis

Statistical analysis was performed using Review Manager 5.4.1 in accordance with the guidelines described in the Cochrane Handbook for Systematic Reviews of Interventions. *P* < 0.05 indicated statistical significance. For dichotomous data, the results were expressed as odds ratios (ORs) with 95% confidence intervals (CIs). For continuous data, the results were pooled for meta-analysis as the mean difference (MD) with 95% CI. When data were reported by different methods or scales, the standardized mean difference with 95% CI was calculated.

Statistical heterogeneity within comparisons was evaluated by Cochran’s Q test and quantified by the *I*-squared (*I*^*2*^) statistic. *I*^*2*^ values < 40% might not be important, values of 30%–60% indicate moderate heterogeneity, values of 50%–90% indicate substantial heterogeneity, and values of 75%–100% indicate considerable heterogeneity [14]. The random-effects method was preferred for calculating summary effect measures because clinical heterogeneity was inevitable. If statistical data were missing from the included studies, we sought further details from the original authors. If participants were lost to follow-up due to failed oocyte retrieval or fertilization, all of them were included in the groups that they were initially assigned to when live birth rate and clinical pregnancy rate were analyzed. Both meta-regression and subgroup analyses were performed to explain potential sources of heterogeneity between studies. We performed the meta-regression analysis on outcomes with ≥10 observations as it was very unlikely that this analysis would produce useful findings unless it included a substantial number of studies. Moreover, subgroup analysis was also conducted to explore the mediating effects of antioxidants according to predefined factors, namely, the type and dose of antioxidants, treatment duration, and characteristics of population, such as age and ovarian reserve. The results of the subgroup analysis were considered only when ≥2 studies were included.

To evaluate the robustness of pooled estimates, sensitivity analysis was performed by removing outlying results, studies with a high risk of bias, or one trial at a time. The potential publication bias of primary outcomes was investigated using Egger’s test and visual inspection of funnel plots.

## Results

### Study selection and characteristics

**FIGURE 1:**
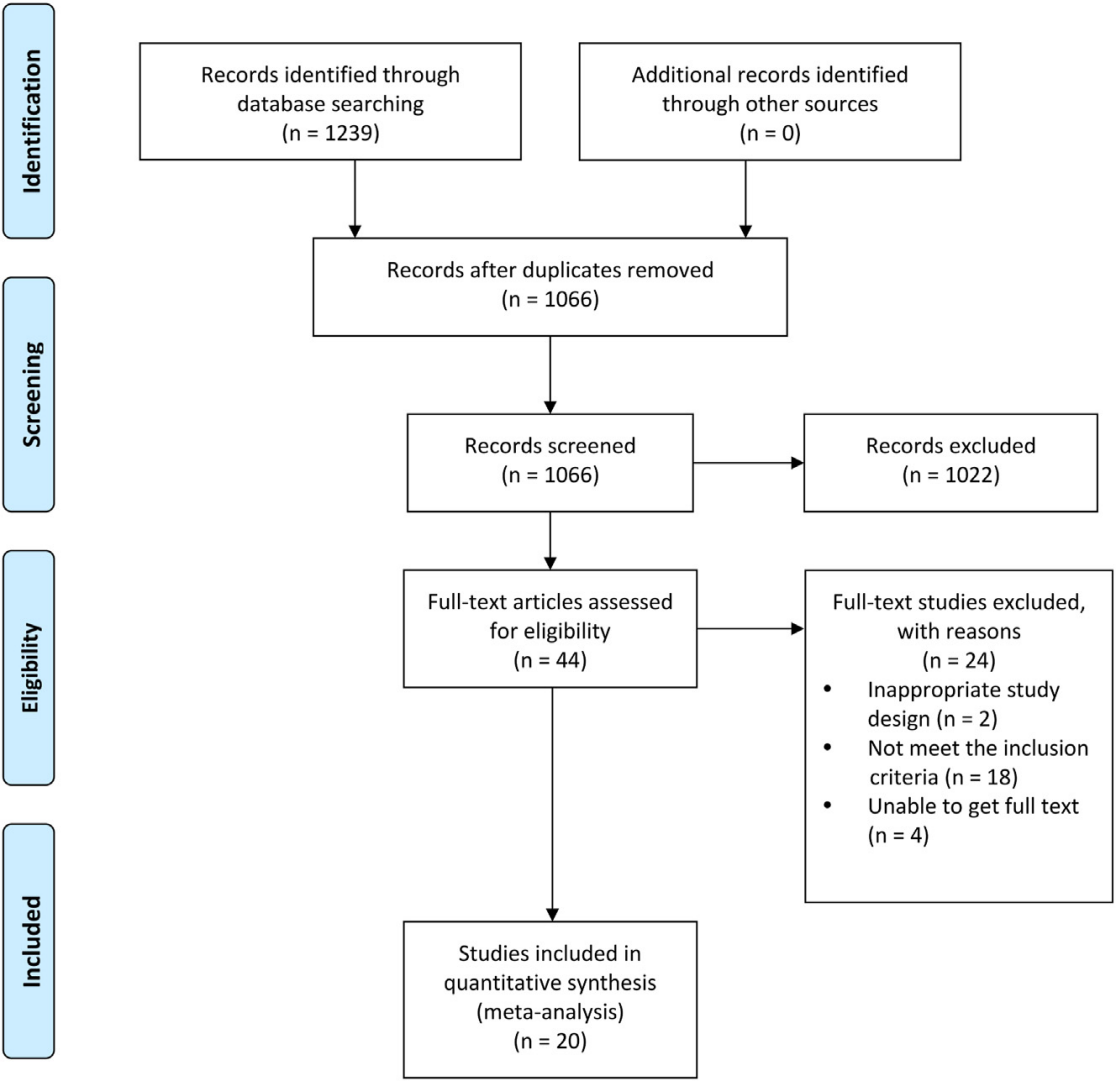
Flow chart of study selection for the systematic review and meta-analysis.

A total of 1239 studies were identified in the preliminary search. After removing 173 duplicates, we assessed the 1066 records by screening the titles and abstracts. Among these, 1022 records were excluded. Forty-four articles were selected for full-text review, 24 of which were excluded for not meeting the inclusion criteria. Finally, 20 RCTs were eligible for meta-analysis. Details of the selection process are shown in the PRISMA flow diagram (Figure 1).

The general characteristics of the included studies are outlined in Table 1 [[15], [16], [17], [18], [19], [20], [21], [22], [23], [24], [25], [26], [27], [28], [29], [30], [31], [32], [33], [34]]. Overall, a total of 20 trials with 2617 participants were included in the analysis. Except for 4 multicenter trials [17,28,33], the rest were all parallel-design RCTs in a single center. Antioxidant treatment involved coenzyme Q10 (CoQ10) (30 mg/d, 200 mg/d, 250 mg/d, 600 mg/d, and 1200 mg/d) [17,19,22,23,25,26,30], melatonin (2–8 mg/d and 16 mg/d) [15,18,20,21,24,34], myo-inositol (4 g/d) [16,29,32], vitamins (vitamin B: 7.8 g/d; vitamin D: 50 mg/d, 600000 IU; and vitamin E: 400 units/d, 0.2 g/d) [27,31,33,35], resveratrol (150 mg/d) [31], and a combination of acetyl-L-carnitine, N-acetyl-L-cysteine, and α-lipoic acid [28]. Treatment duration ranged from a single time to 3 mo. Regarding the different ovarian aging populations, participants could be classified as follows: *1*) advanced age (>35 y) with diminished ovarian reserve [16,[18], [19], [20],^25^,^29^,^30^,^32^]; *2*) advanced age with suboptimal ovarian response [15,17,21,22,24,27,28,31,33]; and *3*) young reproductive age (<35 y) with diminished ovarian reserve [23,26,34]. Among the included trials, the gonadotrophin-releasing hormone antagonist and gonadotrophin-releasing hormone agonist long protocols were the major controlled ovarian stimulation strategies.

**TABLE 1:** Characteristics of trials included in the meta-analysis

Author, year, country	Sample size	Age, y	COS protocol	Antioxidant protocol	Control arm	Outcomes
Rizzo et al. [15], 2010 Italy	Aged women *n* = 65	T: 37.81 ± 2.61 CON: 38.09 ± 1.97	GnRH-a long	Melatonin, 3 mg twice a day, taken orally from the day of GnRH administration.	No treatment	• CPR, MR • Number of retrieved oocytes, MII oocytes and high-quality embryos
Schillaci et al. [16], 2012 Italy	Aged women *n* = 12	T: 36.00 ± 4.50 CON: 36.20 ± 5.40	GnRH-a long	Myo-inositol, 2 g twice a day, taken orally from ≥1 mo before GnRH-a administration.	No treatment	• CPR, MR, cancellation rate • Number of retrieved oocytes • Gn does
Bentov et al. [17], 2014 Italy	Aged women *n* = 39	T: 39.00 ± 0.79 CON: 39.10 ± 0.52	GnRH-a long /GnRH-a short microdose flare	CoQ10, 600 mg/d, taken orally for two months before COS and continued from day 3 of the IVF cycle. Duration of treatment ≤3 cycles if pregnancy did not occur.	Placebo	• LBR, CPR
Valeri et al. [18], 2015 Italy	Aged women *n* = 358	T: >40 CON: >40	GnRH-a short	Melatonin, 5 mg/d, taken orally during IVF protocol.	No treatment	• Mature oocyte rate
Caballero et al. [19], 2016 Argentina	Aged women *n* = 78	T: 37.8 CON: 37.2	—	CoQ10, 600 mg twice a day taken orally for 12 wk.	Placebo	• CPR • Number of MII oocytes
Jahromi et al. [20], 2017 Iran	DOR *n* = 80	T: 35.00 ± 5.10 CON: 35.10 ± 5.10	GnRH-a long	Melatonin, 3 mg/night, taken orally from the 5th day of the menstrual cycle prior to the cycle that was planned for ovarian stimulation.	Placebo	• CPR, MR • Number of MII oocytes, high-quality embryo rate • Gn dose
Fernando et al. [21], 2018 Australia	Aged women *n* = 160	T (4 mg/d): 35.00 ± 4.10 T (8 mg/d): 36.00 ± 4.20 T (16 mg/d): 35.40 ± 4.40 CON: 35.20 ± 4.20	GnRH-A	Melatonin, 2 mg/4 mg/8 mg, twice a day, from day 2 of their cycle until the night before oocyte retrieval.	Placebo	• LBR, CPR • Number of retrieved oocytes, high-quality embryo rate
Taylor et al. [22], 2018 United States	Aged women *n* = 21	36–42	GnRH-a	CoQ10, 125 mg/twice daily, taken orally for 3 mo before the IVF cycle.	Placebo	• Number of retrieved oocytes
Xu et al. [23], 2018 China	DOR *n* = 186	T: 32.50±3.30 CON: 31.92±3.68	GnRH-a short	CoQ10, 200 mg/3 times a day, taken orally for 60 d.	No treatment	• LBR, CPR, MR • Number of retrieved oocytes and high-quality embryos • Gn dose
Espino et al. [24], 2019 Spain	Aged women *n* = 30	T (3 mg): 35.73 ± 3.03 T (6 mg): 36.22 ± 2.71 CON: 36.27 ± 2.08	GnRH-A	Melatonin, 3 mg/6 mg daily, 1 h before sleep from the control ovarian stimulation until the follicular puncture.	No treatment	• CPR • Number of retrieved oocytes and high-quality embryos
Liang [25], 2019 China	DOR *n* = 86	T: 35.42 ± 1.93 CON: 35.78 ± 1.86	GnRH-A	CoQ10, 2 tablets/3 times a day, taken orally for 3 mo.	No treatment	• CPR • Number of retrieved oocytes • Gn dose
Zhang et al. [26], 2019 China	DOR *n* = 185	T: 32.27 ± 3.47 CON: 32.41 ± 2.69	—	CoQ10, 10 mg/3 times a day, taken orally for 3 mo.	No treatment	• CPR • Number of retrieved oocytes
Bezerra Espinola et al. [27], 2021 Italy	Aged women *n* = 120	T: 35.70 ± 6.70 CON: 35.90 ± 3.70	GnRH-A	Vitamin D3, 50 μg (2000 IU)/d, from the day of hCG administration until 14 d after embryo transfer.	No treatment	• CPR, MR • Number of retrieved oocytes, MII oocytes and high-quality embryos • Gn dose
Gardner et al. [28], 2020 Japan	Aged women *n* = 69	36.98 ± 1.87	—	Antioxidants (10 μmol/L) acetyl-L-carnitine, 10 μmol/L N-acetyl-L-cysteine, 5 μmol/L α-lipoic acid in the G-Series media.	No treatment	• CPR
Nazari et al. [29], 2020 Iran	Aged women *n* = 112	T: 37.96 ± 4.64 CON: 37.66 ± 4.35	GnRH-A	Myo-inositol, 4 g/d from 1 mo before the ICSI cycle until the hCG triggering.	No treatment	• CPR, cancellation rate • Number of retrieved oocytes and MII oocytes • Gn dose
Jin et al. [30], 2020 China	Aged women *n* = 92	T: 35.49 ± 7.23 CON: 36.68 ± 7.44	GnRH-a long	CoQ10, 10 mg/3 times a day, taken orally for 3 mo.	No treatment	• CPR • Number of retrieved oocytes and high-quality embryos
Gerli et al. [31], 2021 Italy	Aged women *n* = 90	T: 36.10 ± 0.60 CON: 36.60 ± 0.60	GnRH-A	A resveratrol-based multivitamin supplement (resveratrol 150 mg, folic acid 400 mg, vitamin D 25 mg, vitamin B12 2.5 mg, and vitamin B6 1.4 mg), 2 capsules daily, from 3 mo before the ovarian stimulation until the oocyte retrieval.	No treatment	• LBR, CPR, MR • Number of retrieved oocytes, MII oocytes and high-quality embryos • Gn dose
Mohammadi et al. [32], 2021 Iran	Aged women *n* = 76	T: 35.00 ± 6.91 CON: 36.70 ± 5.60	GnRH-A	Myo-inositol, 4 g/d, 12 wk.	Placebo	• CPR, MR • Number of retrieved oocytes, MII oocytes and high-quality embryos • Gn dose
Somigliana et al. [33], 2021 Italy	*n* = 630	T: 35.00 C: 35.00	—	Vitamin D3, a single administration of oral 600,000 IU, 2–12 wk before the IVF cycle.	Placebo	• LBR, CPR, MR • Number of retrieved oocytes and high-quality embryos • Gn dose
Wang et al. [34]*,* 2022 China	DOR *n* = 128	T: 32.71 ± 3.66 CON: 33.33 ± 4.50	GnRH-A	Melatonin, 10–9 mol/L in the embryo culture medium.	No treatment	• CPR • High-quality embryo rate

Aged women in this table refer to those >35 y old.

Abbreviations: CON, control; COS, controlled ovarian stimulation; CoQ10, coenzyme Q10; CPR, clinical pregnancy rate; DOR, diminished ovarian reserve; Gn, gonadotropin; GnRH-A, gonadotropin-releasing hormone antagonist; GnRH-a, gonadotropin-releasing hormone agonist; hCG, human chorionic gonadotropin; ICSI, intracytoplasmic sperm injection; IVF, in vitro fertilization; LBR, live birth rate; MII, metaphase II; MR, miscarriage rate; POI, premature ovarian insufficiency; T, trial.

### Risk of bias of the included studies

Six trials had low risk of bias across all domains. Seventeen studies (85%) reported random sequence generation. Seven studies (35%) provided allocation concealment. Eight studies (40%) used the double or triple blinding method, and the outcomes of 12 studies (60%) were objective, such that the lack of blinding was unlikely to generate detection bias. Six studies (30%) were judged as having an unclear risk of attrition bias due to participants being lost to follow-up. Ten studies (50%) with registered protocols prior to the trial were considered to have a low risk of reporting bias. Bentov et al. [17] reported the early termination of the trial for embryo safety reasons, which was recognized as possibly causing an overestimation of the effect of the intervention, and the risk of other bias was judged as unclear (Supplementary Figure S1).

### Reproductive outcomes

#### Live birth rate

Seven RCTs with 1224 participants assessed the effectiveness of antioxidant treatment on live birth rate [17,20,21,23,24,31,33]. The analysis was conducted based on the ‘intent-to-treat’ principle. No difference was observed between groups (OR: 1.05; 95% CI: 0.80, 1.37; *P* = 0.74; *I*^*2*^ = 0%; low quality of evidence) (Table 2).

**TABLE 2:** Summary of findings

Outcomes	Anticipated absolute effects^1^ (95% CI)		Relative effect (95% CI)	No. of participants (studies)	Certainty of evidence (GRADE)
	Risk with placebo or no treatment	Risk with antioxidants			
**Reproductive outcomes**					
Live birth rate	262 per 1000	271 per 1000 (221–327)	OR 1.05 (0.80, 1.37)	1224 (7 RCTs)	⊕⊕⊝⊝ Low^2^^,^^3^
Clinical pregnancy rate	267 per 1000	371 per 1000 (301–427)	OR 1.55 (1.18, 2.04)	2218 (21 RCTs)	⊕⊕⊕⊝ Moderate^2^
Miscarriage rate	166 per 1000	137 per 1000 (80–222)	OR 0.80 (0.44, 1.44)	375 (8 RCTs)	⊕⊕⊝⊝ Low^2^^,^^3^
**Quality of oocytes and embryos**					
Number of retrieved oocytes	—	MD 0.98 higher (0.52 higher to 1.44 higher)	—	1722 (15 RCTs)	⊕⊕⊕⊝ Moderate^2^
Number of MII oocytes	—	MD 0.53 higher (0.2 lower to 1.25 higher)	—	1032 (7 RCTs)	⊕⊝⊝⊝ Very low^2^^,^^3^^,^^4^
Number of high-quality embryos	—	MD 0.47 higher (0.16 higher to 0.77 higher)	—	1341 (12 RCTs)	⊕⊕⊕⊝ Moderate^2^
High-quality embryo rate	552 per 1000	741 per 1000 (672–799)	OR 2.32 (1.66, 3.23)	696 (3 RCTs)	⊕⊕⊝⊝ Low^2^^,^^5^
**Ovarian sensitivity**					
Dose of Gn	—	MD 242.71 lower (402.12 lower to 83.3 lower)	—	1208 (8 RCTs)	⊕⊕⊝⊝ Low^2^^,^^4^
**Adverse events**	156 per 1000	190 per 1000 (113–301)	OR 1.27 (0.69, 2.33)	586 (5 RCTs)	⊕⊕⊝⊝ Low^2^^,^^3^

Abbreviations: CI, confidence interval; Gn, gonadotropin; GRADE, Grading of Recommendations Assessment, Development and Evaluation; MD, mean difference; MII, metaphase II; OR, odds ratio; RCT, randomized controlled trial; SMD, standardized mean difference.

1 The risk in the intervention group (and its 95% confidence interval) is based on the assumed risk in the comparison group and the relative effect of the intervention (and its 95% CI).

2 Downgraded one level due to serious risk of bias.

3 Downgraded one level due to serious imprecision: crossing the line of no effect.

4 Downgraded one level due to serious inconsistency with unexplained heterogeneity.

5 Downgraded one level due to serious imprecision: small studies.

#### Clinical pregnancy rate

**FIGURE 2:**
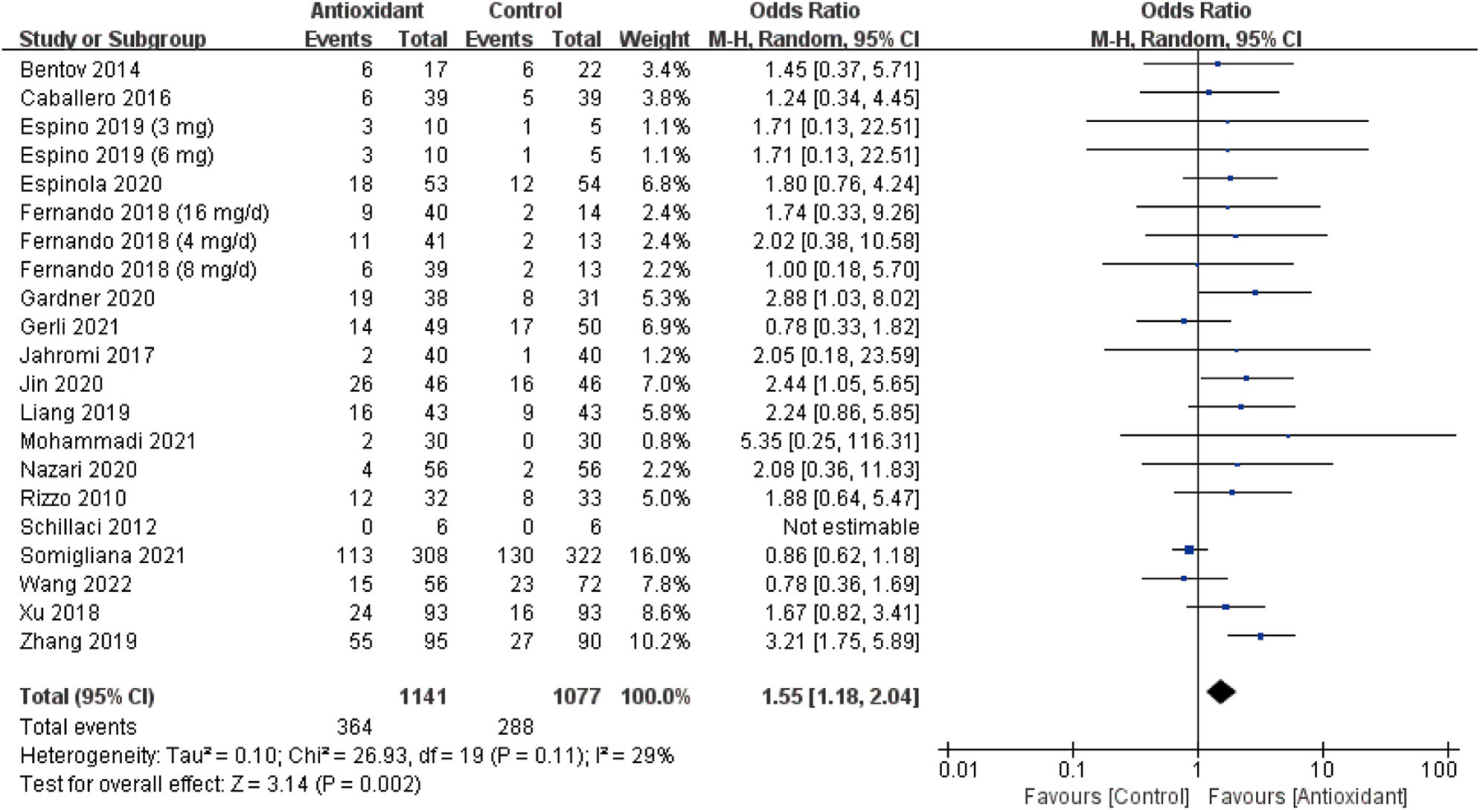
Forest plots of clinical pregnancy rate. Antioxidant versus placebo or no treatment (control). CI, confidence interval; M-H, Mantel–Haenszel.

Twenty RCTs with 2218 participants evaluated the effects of antioxidants on clinical pregnancy rate [[15], [16], [17],[19], [20], [21],[23], [24], [25], [26], [27], [28], [29], [30], [31], [32], [33], [34],^36^]. According to the ‘intent-to-treat’ principle, we found that the clinical pregnancy rate in the antioxidative group was significantly higher than that in the control group (OR: 1.55; 95% CI: 1.18, 2.04; *P* = 0.002; *I*^*2*^ = 29%; moderate quality of evidence) (Figure 2, Table 2).

#### Miscarriage rate

Eight trials reported the outcomes of spontaneous abortion [15,16,20,23,27,[31], [32], [33]]. Pooled data indicated that antioxidants exhibited no advantages on miscarriage rate (OR: 0.80; 95% CI: 0.44, 1.44; *P* = 0.45; *I*^*2*^ = 0%; moderate quality of evidence) (Table 2).

### Quality of oocyte and embryo

#### Number of retrieved oocytes

**FIGURE 3:**
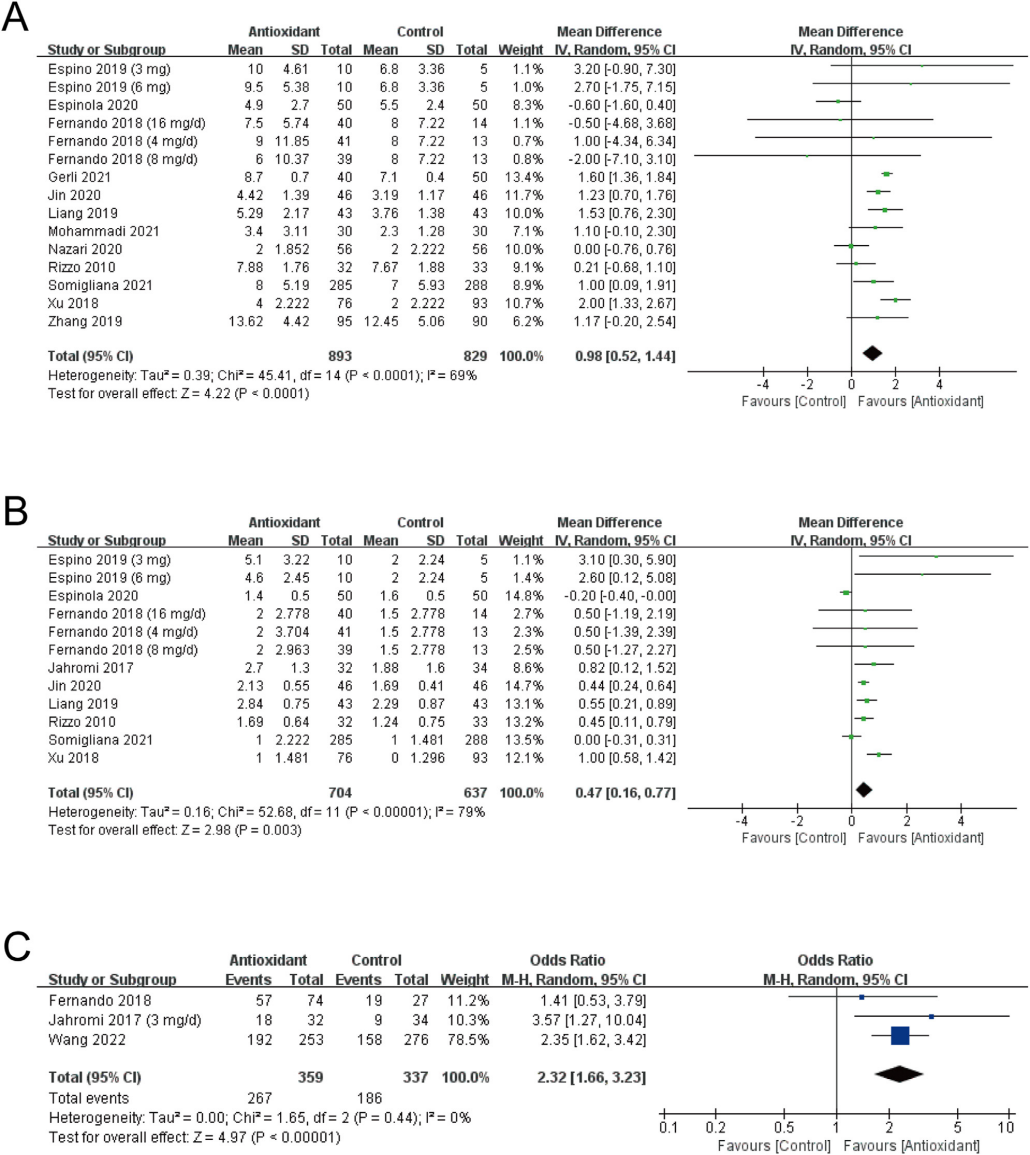
Forest plots of oocyte and embryo quality. Antioxidant versus placebo or no treatment (control): (A) number of retrieved oocytes, (B) number of high-quality embryos and (C) high-quality embryo rate. CI, confidence interval; IV, inverse variance; M-H, Mantel–Haenszel; SD, standard deviation.

The number of retrieved oocytes was evaluated in 13 RCTs with 1734 participants. One RCT was not included in the meta-analysis due to unavailable data, in which no difference was found between the myo-inositol group and the control group [16]. The results of the meta-analysis showed that antioxidants significantly increased the number of retrieved oocytes (MD: 0.98; 95% CI: 0.52, 1.44; *P* < 0.0001; *I*^*2*^ = 69%; moderate quality of evidence) (Figure 3A, Table 2) [15,21,[23], [24], [25], [26], [27],[29], [30], [31], [32], [33]].

#### Number of MII oocytes

Ten RCTs with 1523 women provided information on the number of MII oocytes. Three trials were not included in the meta-analysis because of insufficient data [18,22,29]. No advantages of antioxidant treatment were found in the studies of Nazari et al. [29] and Taylor et al. [22], whereas the results of Valeri et al. [18] supported the benefits of antioxidants in promoting oocyte maturation (48.2% compared with 35.0%, *P* = 0.008). Pooled data of the remaining studies revealed no statistically significant difference between groups (MD: 0.53; 95% CI: −0.20, 1.25; *P* = 0.16; *I*^*2*^ = 93%; very low quality of evidence) [15,19,20,27,[31], [32], [33]] (Table 2).

#### Number of high-quality embryos

Twelve RCTs with 1939 women investigated the role of antioxidants in embryo quality [15,18,20,21,[23], [24], [25],^27^,^29^,^30^,^33^,^34^]. The results of 9 trials were presented as the number of high-quality embryos, and 5 studies calculated the proportion of good embryos. Overall, treatment with antioxidants led to more high-quality embryos. Pooled data were shown as follows: *1*) number of high-quality embryos: MD: 0.47; 95% CI: 0.16, 0.77; *P* = 0.003; *I*^*2*^ = 79%; moderate quality of evidence) (Figure 3B, Table 2); *2*) high-quality embryo rate (proportion of high-quality embryos): OR: 2.32; 95% CI: 1.66, 3.23; *P* < 0.00001; *I*^*2*^ = 0%; low quality of evidence (Figure 3C, Table 2). The studies by Valeri et al. [18] and Nazari et al. [29] were not included in the analysis because of unavailable data, which supported the benefits of antioxidants in embryo quality (Valeri et al. [18]: 67.2% compared with 36.5%, *P* < 0.001; Nazari et al. [29]: 47.5% compared with 30.4%, *P* = 0.0045).

### Dose of gonadotrophin

**FIGURE 4:**
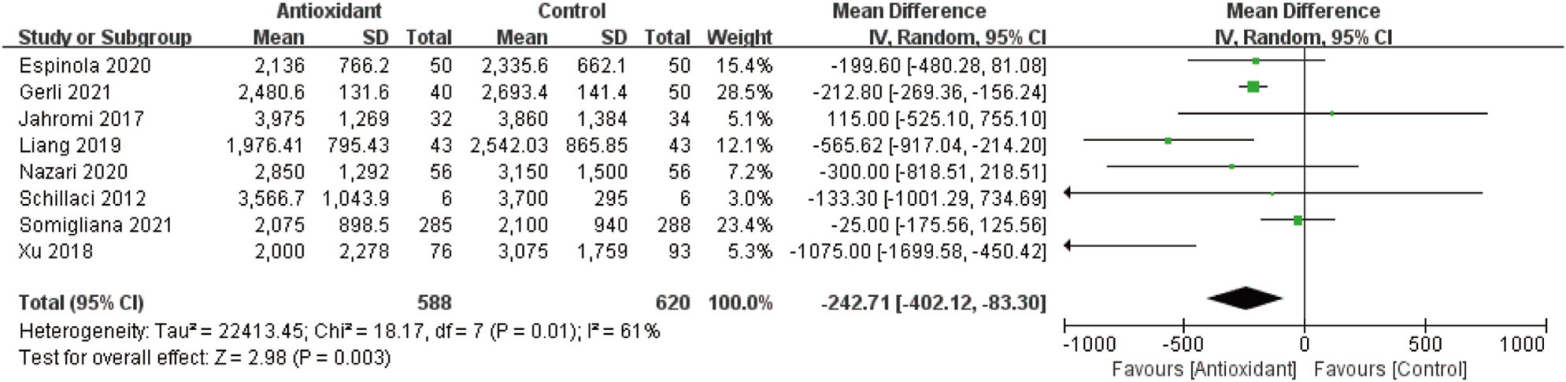
Forest plots of gonadotropin dose. Antioxidant versus placebo or no treatment (control). CI, confidence interval; IV, inverse variance; SD, standard deviation.

Eight studies with 1208 women provided data on the dose of gonadotropin [16,20,23,25,27,29,31,33]. The results revealed that antioxidant treatment significantly reduced the total dose of gonadotropin required during controlled ovarian stimulation (MD: −242.71; 95% CI: −402.12, −83.30; *P* = 0.003; *I*^*2*^ = 61%; low quality of evidence) (Figure 4, Table 2).

### Adverse events

Five RCTs with 678 participants mentioned adverse events [20,21,23,30,31], including pregnancy complications (congenital missing kidney, low birth weight, and preterm birth) and minor side effects (headache, dizziness, nausea, and vomiting). There was no evidence to suggest an association between antioxidants and adverse events (OR: 1.27; 95% CI: 0.69, 2.33; *P* = 0.45; *I*^*2*^ = 2%; low quality of evidence) (Table 2).

### Subgroup analysis

#### Subgrouped by type of antioxidant

In terms of clinical pregnancy rate, when compared with placebo/no treatment, women treated with CoQ10 were more likely to become pregnant. Improvements were also observed with other antioxidants, including melatonin, myo-inositol, vitamins, resveratrol, acetyl-L-carnitine, N-acetyl-L-cysteine, and α-lipoic acid, but these improvements were not statistically significant. Consistent with the optimal suggestions for clinical pregnancy rate, CoQ10 led to more collected oocytes, whereas treatment with melatonin, myo-inositol, and vitamins had a tendency to bring benefits. Regarding the number of high-quality embryos, both CoQ10 and melatonin were more effective in promoting the quality of embryos than placebo/no treatment; however, no effects were observed between the vitamin D3 and control arms. With respect to gonadotropin dose, CoQ10 was significantly associated with better ovarian sensitivity due to less gonadotropin; however, small improvements were reported from reducing the dose of gonadotropin after treatment with melatonin, myo-inositol, and vitamins (Table 3).

**TABLE 3:** Subgroup analysis by type of antioxidant

Subgroup	No. of studies	No. of women	Effect estimate OR/MD (95% CI)	*I*^*2*^	*P*
**Clinical pregnancy rate (%)**					
CoQ10	6	666	2.22 (1.57, 3.14)	0%	<0.00001
Melatonin	8	463	1.24 (0.75, 2.03)	0%	0.40
Myo-inositol	3	184	2.61 (0.57, 11.88)	0%	0.21
Vitamins	2	737	1.11 (0.55, 2.23)	61%	0.77
Combined antioxidants	2	168	1.45 (0.40, 5.21)	73%	0.57
**Number of retrieved oocytes**					
CoQ10	4	532	1.51 (1.13, 1.89)	11%	<0.00001
Melatonin	3	255	0.35 (−0.47, 1.16)	0%	0.40
Myo-inositol	2	172	0.45 (−0.61, 1.51)	56%	0.41
Vitamins	2	673	0.21 (−1.35, 1.78)	81%	0.79
Combined antioxidants	1	90	1.60 (1.36, 1.84)	—	<0.00001
**Number of high-quality embryos**					
CoQ10	3	347	0.62 (0.32, 0.92)	64%	<0.0001
Melatonin	4	321	0.64 (0.26, 1.02)	10%	0.0010
Vitamins	2	673	−0.14 (−0.32, 0.04)	13%	0.14
**Gonadotropin dose**					
CoQ10	2	255	−752.19 (−1233.18, −271.19)	48%	0.002
Melatonin	1	66	115.00 (−525.10, 755.10)	—	0.72
Myo-inositol	2	124	−256.16 (−701.29, 188.98)	0%	0.26
Vitamins	2	673	−70.47 (−220.65, 79.71)	13%	0.36
Combined antioxidants	1	90	−212.80 (−269.36, −156.24)	—	<0.00001

Abbreviations: CI, confidence interval; CoQ10, coenzyme Q10; MD, mean difference; OR, odds ratio.

#### Subgrouped by treatment duration

Regarding clinical pregnancy rate, treatment during controlled ovarian stimulation or 3 mo before controlled ovarian stimulation contributed to higher clinical pregnancy rate (Supplementary Table S2). Relating to the quality of oocytes and embryos, improvements in the number of retrieved oocytes and high-quality embryos were more pronounced when there was pretreatment beginning 3 mo before controlled ovarian stimulation (Supplementary Table S3). Regarding ovarian sensitivity, beginning administration 3 mo before controlled ovarian stimulation significantly decreased the dose of gonadotropin. Due to the limited number of studies (only 1 trial), the effect of beginning antioxidant treatment 2 mo before controlled ovarian stimulation was unclear (Supplementary Table S4).

#### Subgrouped by ovarian aging population

The subgroup analysis of the ovarian aging population was performed by age and ovarian reserve of participants. The results demonstrated that antioxidants had greater effects on clinical pregnancy rate in women aged >35 y with diminished ovarian reserve (Supplementary Table S2). Similarly, those women also gained more high-quality embryos with antioxidant treatment. In addition, antioxidants also contributed to more retrieved oocytes in women with diminished ovarian reserve and significantly improved ovarian sensitivity in women of advanced age (Supplementary Table S3 and S4).

#### Treatment regimens of specific antioxidants

Given that CoQ10 showed greater benefits in all of the outcomes and that melatonin contributed to better embryo quality (*P* < 0.05), subgroup analysis would produce useful findings only with an adequate number of studies. Further analysis of CoQ10 in clinical pregnancy rate and melatonin in number of high-quality embryos were conducted by dose, treatment duration, and ovarian population to seek optimal treatment regimens. The results indicated that 30 mg CoQ10/d for 3 mo before the controlled ovarian stimulation cycle significantly improved clinical pregnancy rate, and women with diminished ovarian reserve had higher clinical pregnancy rate after CoQ10 treatment, especially those of young reproductive age (Table 4). With regard to melatonin, low dose (<5 mg/d) was the optimal dose when compared with others, and melatonin could improve the quality of embryos in women of advanced age. Among them, in women with suboptimal ovarian response, intervention during the controlled ovarian stimulation cycle effectively led to more high-quality embryos, whereas for those with diminished ovarian reserve, the duration may be longer (1 mo) (Supplementary Table S5). Taking the results of the subgroup analysis on CoQ10 and melatonin together, we found a dose-dependent relationship between antioxidant treatment and fertility outcomes, with a tendency toward a better effect with a lower dose.

**TABLE 4:** Subgroup analysis of CoQ10 on clinical pregnancy rate

Subgroup	No. of studies	No. of women	Effect estimate OR (95% CI)	*I*^*2*^	*P*
**Dose**					
30 mg/d	3	363	2.76 (1.78, 4.28)	0%	<0.00001
600 mg/d	2	225	1.62 (0.86, 3.05)	0%	0.13
1200 mg/d	1	78	1.24 (0.34, 4.45)	—	0.75
**Treatment duration**					
2 mo before the COS	2	225	1.62 (0.86, 3.05)	0%	0.13
3 mo before the COS	4	441	2.54 (1.68, 3.84)	0%	<0.0001
**Population**					
>35 y old with diminished ovarian reserve	3	356	2.07 (1.17, 3.65)	0%	0.01
>35 y old with suboptimal ovarian response	1	39	1.38 (0.29, 6.58)	—	0.68
<35 y old with diminished ovarian reserve	2	371	2.38 (1.26, 4.50)	46%	0.007

Abbreviations: CI, confidence interval; CoQ10, coenzyme Q10; COS, controlled ovarian stimulation; OR, odds ratio.

### Meta-regression analysis

Meta-regression analysis was conducted via a random-effects model. The results revealed that treatment duration (*P* = 0.022) and antioxidant type (*P* = 0.033) were important covariates for the number of retrieved oocytes and high-quality embryos, respectively, accounting for the sources of heterogeneity. However, no significant associations were observed in clinical pregnancy rate, number of MII oocytes, and dose of gonadotropin (Supplementary Table S6).

### Sensitivity analysis and publication bias

**FIGURE 5:**
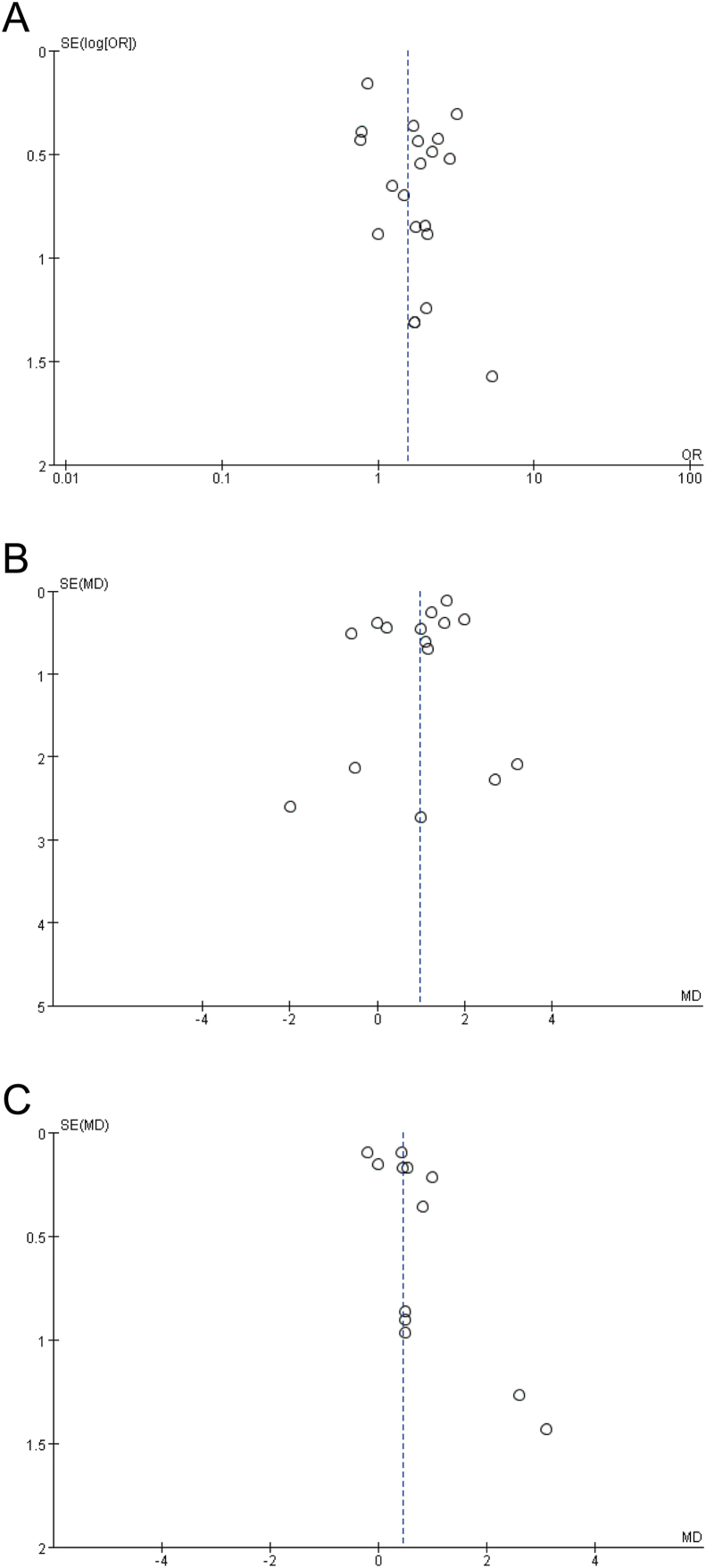
Funnel plots of clinical pregnancy rate (A), number of retrieved oocytes (B), and number of high-quality embryos (C). MD, mean difference; OR, odds ratio; SE, standard error.

All of the results were stable, with unchanged estimates after sensitivity analysis. Egger’s test was conducted to examine primary outcomes. Given that the assessment can be underpowered with small numbers of studies (<10), only outcomes on clinical pregnancy rate, retrieved oocytes, and embryo quality were considered. As presented in Figure 5, no asymmetries were found in the funnel plots, and this was further supported by the result of Egger’s test (*P*_clinical pregnancy rate_ = 0.051; *P*_*retrieved oocytes*_ = 0.132; *P*_*high-quality embryos*_ = 0.099).

## Discussion

To our knowledge, this study is the first meta-analysis to provide a comprehensive analysis of data on currently available antioxidants used in women with ovarian aging during IVF. The collective evidence indicated that antioxidants not only significantly increased the number of retrieved oocytes and high-quality embryos but also reduced dose of gonadotropin, leading to a higher clinical pregnancy rate. However, the effects on the live birth rate were unclear. According to our subgroup analysis, a dose-dependent relationship between antioxidant treatment and reproductive outcomes was also found in the analysis, namely, a low dose seemed to result in greater improvements. Hence, a low dose should be given priority in the clinical application of antioxidants. In terms of different types of antioxidants, CoQ10 tended to be more effective than melatonin, myo-inositol, and vitamins. We further performed subgroup analysis on CoQ10 to provide optimal treatment protocols. For CoQ10, 30 mg/d for 3 mo before the controlled ovarian stimulation cycle was the optimal regimen, and women with diminished ovarian reserve were the most appropriate population to benefit from CoQ10 treatment, especially those aged <35 y.

Given the characteristics of ovarian aging, along with the phenomenon of delayed birth, it has been considered a vital cause of infertility and population aging, leading to many deleterious consequences for social development and human well-being [37]. Although antioxidants have been widely used to improve IVF outcomes, few clinical studies have evaluated the role of antioxidants in this population. Previous studies fail to provide useful recommendations for clinical application because they did not assess the effects of specific antioxidant and focus on the general infertile population, with the characteristics of different antioxidants and infertile population largely overlooked [38]. In contrast, we concretized the study population as ovarian aging patients, because it is a vital factor of infertility, and broadened the scope of observed outcomes. In our study, not only did we pay attention to the characteristics of different antioxidants (type, dose, and duration) and participants (age and ovarian reserve), but we also observed reproductive outcomes concerning the quality of oocyte and embryo, which enriches the role of antioxidants in promoting fertility. Our results demonstrated that the application of antioxidants significantly improved reproductive outcomes by increasing the number of retrieved oocytes and high-quality embryos and reducing the dose of gonadotropin. Notably, considering drug-related characteristics as well as unique sensitivities to the treatment in participants with different ages and ovarian reserve, we performed detailed subgroup analysis and meta-regression analysis based on types of antioxidants, treatment duration, and population characteristics to explain the potential sources of heterogeneity and moderator variables of overall effects. According to meta-regression analysis, we found strong correlations between treatment duration and the number of retrieved oocytes as well as antioxidant type and high-quality embryos, indicating that antioxidant type and treatment duration are the major variables for heterogeneity and overall effects.

CoQ10, as a lipid-soluble benzoquinone present in the cells of almost all aerobic organisms, is an essential electron transporter in the mitochondrial respiratory chain [39]. Several observational studies have demonstrated a tissue-specific decline in CoQ10 concentrations with age [40]. A recently published cross-sectional study also demonstrated that the serum CoQ10/total cholesterol ratio was inversely associated with premature ovarian insufficiency [41]. Women aged <41 y with higher CoQ10 concentrations in their follicular fluid have better embryo morphogenetic parameters and higher pregnancy rates [42]. The above evidence indicates that CoQ10 deficiency is significantly associated with ovarian aging and infertility. Emerging evidence has shown that CoQ10 supplementation leads to improvement of ovarian reserve and oocyte quality by resulting in lower rates of apoptosis and meiotic abnormalities as well as better mitochondrial function and reproductive performance [39]. Pooled data from our analysis also supported CoQ10 as a promising strategy for rescuing defects generated by ovarian aging. Based on our subgroup analysis, we found CoQ10 to be more effective among antioxidants included in the analysis, because it exhibited more advantages when compared with placebo or no treatment, whereas others showed little effects without statistical significance. To seek the optimal regimen and provide evidence-based suggestions for clinical practice, we further performed subgroup analysis by dose, treatment duration, and participant characteristics. The optimal recommendation for CoQ10 in improving clinical pregnancy rate was 30 mg/d for 3 mo before the controlled ovarian stimulation cycle, and women with diminished ovarian reserve were the most appropriate population to benefit from the treatment, especially those of young reproductive age. However, based on previous studies [23,43] and our own findings, the effect of CoQ10 treatment on the live birth rate is still unclear; hence, recommending CoQ10 for women might still be controversial, and more trials with large sample sizes and high-quality methodology are necessary to confirm the effects.

Given the results of the subgroup analysis based on duration, the optimal duration was 3 mo before controlled ovarian stimulation due to more improvements in clinical pregnancy rate. It takes ∼85 d for primary follicles to ovulate [44]. A long course can ensure that the intervention works throughout the folliculogenesis process to improve ovarian function. In addition, a short treatment length may fail to achieve the required amount of stimulation, which is likely to weaken the potentially positive effect of intervention.

Women are born with a finite follicle pool that will go through constant decline without renewing, and the depletion process is accelerated at ∼35 y of age coupled with a decrease in oocyte quality, leading to a gradual loss of fertility. It has been demonstrated that even in young women with diminished ovarian reserve, the chances of achieving high-quality embryos and successful pregnancy in IVF are much greater than those of older women, despite similar numbers of eggs being obtained. In terms of the ovarian aging population with different ages and ovarian reserve, our evidence suggested that CoQ10 significantly improved the fertility of women with diminished ovarian reserve, and the younger the woman, the more obvious the effect. With the delay in childbearing, aged women with poor ovarian reserve become more prevalent, constituting 55% of the poor ovarian response population in some centers [45]. The dual negative effect of a reduced ovarian reserve (quantity) as well as an age-related increase in aneuploidy (quality) makes this category of patients difficult to handle [46]. Therefore, CoQ10 supplementation could be considered when standard treatment protocols fail to obtain satisfactory results.

To the best of our knowledge, this study represents the first comprehensive analysis of data on currently available antioxidant strategies for ovarian aging. We evaluated the effectiveness and safety of antioxidants in the ovarian aging population and provided evidence-based recommendations for CoQ10 in promoting reproductive outcomes from the aspects of dose, duration, and targeted population, thus contributing to prolonging reproductive lifespan and helping to prevent ovarian aging in clinical practice. In addition, our research was registered with PROSPERO and strictly performed in accordance with the PRISMA statement. All the procedures were faithfully executed, and the quality of the methodology was high.

However, several limitations should also be considered. First, the quality of some results was limited and should be interpreted with caution. To explain the potential source of heterogeneity, detailed subgroup analysis and meta-regression analysis were performed, and we regarded antioxidant type and treatment duration as the main causes. However, there is still unexplainable heterogeneity derived from clinical and methodological differences between studies. Second, we were unable to ascertain treatment regimens for other antioxidants and the administration methods (oral or added for oocyte collection and embryo culture in vitro) due to the limited number of studies, which highlights the lack of scholarly attention to the management of ovarian aging. Third, given the inadequate number of trials and small sample size in certain subgroups, the data might have been insufficient to detect important differences, thus limiting our inferences. Therefore, more studies are needed for further assessment.

At present, many challenges remain, such as treatment regimens and administration methods, and hinder its application. Future research requires interdisciplinary research among aging, reproduction, and antioxidants to explore the mechanisms driving ovarian aging and further clarify the exact role of antioxidants in protecting ovarian function during aging, thus further standardizing the clinical application of antioxidants in ovarian aging management. In addition, it is highly important to observe the safety profile when assessing the role of antioxidants in promoting reproductive health during ovarian aging. Future work should pay more attention to adverse effects during treatment as well as neonatal outcomes and risk of birth defects.

In conclusion, our study suggests that antioxidant therapy is an effective and safe complementary strategy during IVF for women with ovarian aging. Among them, treatment with CoQ10 is a promising choice for rescuing the decline in fertility caused by ovarian aging. The optimal treatment regimen for CoQ10 was 30 mg/d for 3 mo before the controlled ovarian stimulation cycle, and women with diminished ovarian reserve clearly benefited from the treatment, especially those of young reproductive age. In addition, there may be a dose-dependent relationship between antioxidant treatment and reproductive outcomes, namely, a low dose tends to bring more benefits. Hence, appropriate antioxidant treatment should be offered from a low dose according to the patient’s age and ovarian reserve. In general, the quality of comparative evidence is not high, and further evaluation in RCTs with larger sample sizes and rigorous designs is warranted to confirm the effects of antioxidants on reproductive outcomes in ovarian aging.

## Author contributions

The authors’ responsibilities were as follows – YS, MW: conceived the study and designed the study protocol; YS, NS, RH: performed study selection, data extraction, quality assessment, and results interpretation; YS: performed the statistical analysis and drafted the paper; and all authors: read and approved the final manuscript.

## Conflicts of interest

The authors report no conflicts of interest.

## Funding

This work is supported by the Project funded by National Natural Science Foundation of China (82305305), China Postdoctoral Science Foundation (2023M733981), and Natural Science Foundation of Hunan Province (2023JJ40799).

## Data availability

The corresponding author will provide data and other materials used in this review upon reasonable request.
