# Supplementary material for: Antioxidants and Fertility in Women with Ovarian Aging: A Systematic Review and Meta-Analysis
Source: Adv Nutr. 2024 Jul 15;15(8):100273. doi: 10.1016/j.advnut.2024.100273 (PMC11345374; doi:10.1016/j.advnut.2024.100273)
Supplement: multimedia component [file mmc1.docx]

**Supplementary Information**

**Antioxidants and fertility in women with ovarian ageing: A systematic review and meta-analysis**

Yujie Shang, Nannan Song, Ruohan He, Minghua Wu

Corresponding author:

Minghua Wu

School of Basic Medical Sciences, Central South University

110 Xiang-Ya Road, Changsha, Hunan 410078, China

E-mail: wuminghua554@aliyun.com

Tel: 86-731-4805383 Fax: 86-731-4805383

**Content**

**Supplementary Table S1.** Search strategy for PubMed (results from September 12,2023)

**Supplementary Table S2.** Subgroup analyses on clinical pregnancy rate

**Supplementary Table S3.** Subgroup analyses for oocytes and embryos outcomes

**Supplementary Table S4.** Subgroup analyses for gonadotropin does

**Supplementary Table S5.** Subgroup analysis of melatonin on number of high-quality embryos

**Supplementary Table S6.** Meta-regression analyses of outcomes and moderator variables

**Supplementary Figure S1.** Risk of bias assessment of included studies

**Supplementary Table S1.** Search strategy for PubMed (results from September 12,2023)

| **Search** |  | **Results** |
| --- | --- | --- |
| #1 | "antioxidants"[MeSH Terms] OR "free radical scavengers"[MeSH Terms] OR "antioxidants"[Title/Abstract] OR "antioxidants"[Title/Abstract] OR "vitamins"[Title/Abstract] OR "ascorbic acid"[Title/Abstract] OR "dehydroascorbic acid"[Title/Abstract] OR "vitamin a"[Title/Abstract] OR "vitamin e"[Title/Abstract] OR "vitamin u"[Title/Abstract] OR "alpha-tocopherol"[Title/Abstract] OR "beta carotene"[Title/Abstract] OR "beta-tocopherol"[Title/Abstract] OR "gamma-tocopherol"[Title/Abstract] OR "vitamin b 12"[Title/Abstract] OR "Vitamin-B-12-Therapeutic-Use"[Title/Abstract] OR "vitamin b6"[Title/Abstract] OR "vitamin c"[Title/Abstract] OR "vitamin d"[Title/Abstract] OR "Vitamin"[Title/Abstract] OR "Zinc"[Title/Abstract] OR "Selenium"[Title/Abstract] OR "glutathione peroxidase"[Title/Abstract] OR "folic acid"[Title/Abstract] OR "Ubiquinone"[Title/Abstract] OR "coenzyme q10"[Title/Abstract] OR "Carnitine"[Title/Abstract] OR "multivitamin"[Title/Abstract] OR "carotenoid"[Title/Abstract] OR "astaxanthin"[Title/Abstract] OR "lycopene"[Title/Abstract] OR "betacarotene"[Title/Abstract] OR "beta carotene"[Title/Abstract] OR "ascorbic acid"[Title/Abstract] OR "n-acetylcysteine"[Title/Abstract] OR "Acetylcysteine"[Title/Abstract] OR "alpha-tocopherol"[Title/Abstract] OR "fish oil"[Title/Abstract] OR "fatty acids"[Title/Abstract] OR "fish oils"[Title/Abstract] OR "cod liver oil"[Title/Abstract] OR "fatty acids"[Title/Abstract] OR "fatty acids omega 3"[Title/Abstract] OR "plant oils"[Title/Abstract] OR "flavonoid"[Title/Abstract] OR "Quercetin"[Title/Abstract] OR "Flavonoids"[Title/Abstract] OR "riboflavin"[Title/Abstract] OR "pycnogenol"[Title/Abstract] OR "lutein"[Title/Abstract] OR "lipoic acid"[Title/Abstract] OR "n acetyl cysteine"[Title/Abstract] OR "melatonin"[Title/Abstract] OR "dietary supplement"[Title/Abstract] OR "nutritional supplement"[Title/Abstract] OR "micronutrient"[Title/Abstract] OR "Nutraceuticals"[Title/Abstract] OR "Chromium"[Title/Abstract] OR "chromax"[Title/Abstract] OR "myoinositol"[Title/Abstract] OR "mesoinositol"[Title/Abstract] OR "Inositol"[Title/Abstract] OR "Pentoxifylline"[Title/Abstract] | 983253 |
| #2 | "fertilization in vitro"[MeSH Terms] OR "in vitro fertilization"[Title/Abstract] OR "in vitro fertilizations"[Title/Abstract] OR "test tube fertilization"[Title/Abstract] OR "fertilization test tube"[Title/Abstract] OR (("fertilisability"[All Fields] OR "fertilisable"[All Fields] OR "fertilisation"[All Fields] OR "Fertilization"[MeSH Terms] OR "Fertilization"[All Fields] OR "fertilisations"[All Fields] OR "fertilise"[All Fields] OR "fertilised"[All Fields] OR "fertilisers"[All Fields] OR "fertilizers"[Pharmacological Action] OR "fertilizers"[MeSH Terms] OR "fertilizers"[All Fields] OR "fertilises"[All Fields] OR "fertilising"[All Fields] OR "Fertilizations"[All Fields] OR "fertilize"[All Fields] OR "fertilized"[All Fields] OR "fertiliser"[All Fields] OR "fertilizer"[All Fields] OR "fertilizes"[All Fields] OR "fertilizing"[All Fields]) AND "Test-Tube"[Title/Abstract]) OR "test tube fertilization"[Title/Abstract] OR ("Test-Tube"[All Fields] AND "Fertilizations"[Title/Abstract]) OR "fertilizations in vitro"[Title/Abstract] OR "test tube babies"[Title/Abstract] OR (("baby s"[All Fields] OR "babys"[All Fields] OR "infant"[MeSH Terms] OR "infant"[All Fields] OR "Babies"[All Fields]) AND "Test-Tube"[Title/Abstract]) OR (("infant, newborn"[MeSH Terms] OR ("infant"[All Fields] AND "newborn"[All Fields]) OR "newborn infant"[All Fields] OR "Baby"[All Fields] OR "infant"[MeSH Terms] OR "infant"[All Fields]) AND "Test-Tube"[Title/Abstract]) OR "test tube babies"[Title/Abstract] OR "test tube baby"[Title/Abstract] OR ("reproductive techniques, assisted"[MeSH Terms] OR "assisted reproductive technique"[Title/Abstract] OR "reproductive technique assisted"[Title/Abstract] OR (("methods"[MeSH Subheading] OR "methods"[All Fields] OR "Techniques"[All Fields] OR "methods"[MeSH Terms] OR "Technique"[All Fields] OR "technique s"[All Fields]) AND "assisted reproductive"[Title/Abstract]) OR (("methods"[MeSH Subheading] OR "methods"[All Fields] OR "Techniques"[All Fields] OR "methods"[MeSH Terms] OR "Technique"[All Fields] OR "technique s"[All Fields]) AND "assisted reproductive"[Title/Abstract]) OR "assisted reproductive technics"[Title/Abstract] OR "assisted reproductive technic"[Title/Abstract] OR ((("reproduction"[MeSH Terms] OR "reproduction"[All Fields] OR "reproductions"[All Fields] OR "Reproductive"[All Fields] OR "reproductively"[All Fields] OR "reproductives"[All Fields] OR "reproductivity"[All Fields]) AND ("Technic"[All Fields] OR "Technics"[All Fields])) AND "Assisted"[Title/Abstract]) OR ((("reproduction"[MeSH Terms] OR "reproduction"[All Fields] OR "reproductions"[All Fields] OR "Reproductive"[All Fields] OR "reproductively"[All Fields] OR "reproductives"[All Fields] OR "reproductivity"[All Fields]) AND ("Technic"[All Fields] OR "Technics"[All Fields])) AND "Assisted"[Title/Abstract]) OR (("Technic"[All Fields] OR "Technics"[All Fields]) AND "assisted reproductive"[Title/Abstract]) OR (("Technic"[All Fields] OR "Technics"[All Fields]) AND "assisted reproductive"[Title/Abstract]) OR "assisted reproductive techniques"[Title/Abstract] OR (("reproduction"[MeSH Terms] OR "reproduction"[All Fields] OR "reproductions"[All Fields] OR "Reproductive"[All Fields] OR "reproductively"[All Fields] OR "reproductives"[All Fields] OR "reproductivity"[All Fields]) AND "technology assisted"[Title/Abstract]) OR "assisted reproductive technologies"[Title/Abstract] OR "assisted reproductive technology"[Title/Abstract] OR "reproductive technologies assisted"[Title/Abstract] OR "technologies assisted reproductive"[Title/Abstract] OR (("Technology"[MeSH Terms] OR "Technology"[All Fields] OR "Technologies"[All Fields] OR "technology s"[All Fields]) AND "assisted reproductive"[Title/Abstract])) OR ("IVF"[Title/Abstract] OR "ICSI"[Title/Abstract] OR "intra cytoplasmic sperm injection"[Title/Abstract] OR "intracytoplasmic sperm injection"[Title/Abstract] OR "fertilization in vitro"[MeSH Terms]) OR ("subfertility"[Title/Abstract] OR "infertility"[Title/Abstract] OR "sterility reproductive"[Title/Abstract] OR "reproductive sterility"[Title/Abstract] OR "Sub-Fertility"[Title/Abstract]) OR ("decreased ovarian reserve"[Title/Abstract] OR "diminished ovarian reserve"[Title/Abstract]) OR ("ovarian ageing"[Title/Abstract] OR "ovarian aging"[Title/Abstract] OR "ovarian dysfunction"[Title/Abstract] OR "ovarian senescence"[Title/Abstract]) OR ("primary ovarian insufficiency"[MeSH Terms] OR "primary ovarian insufficiency"[Title/Abstract] OR "ovarian insufficiency primary"[Title/Abstract] OR "ovarian failure premature"[Title/Abstract] OR "premature ovarian failure"[Title/Abstract] OR "gonadotropin resistant ovary syndrome"[Title/Abstract] OR "gonadotropin resistant ovary syndrome"[Title/Abstract] OR "resistant ovary syndrome"[Title/Abstract] OR ((("Hypergonadotropic"[All Fields] OR "hypergonadotropism"[All Fields]) AND ("Ovarian"[All Fields] OR "ovarians"[All Fields]) AND ("Failure"[All Fields] OR "failures"[All Fields])) AND "X-Linked"[Title/Abstract]) OR ((("Hypergonadotropic"[All Fields] OR "hypergonadotropism"[All Fields]) AND ("Ovarian"[All Fields] OR "ovarians"[All Fields]) AND ("Failure"[All Fields] OR "failures"[All Fields])) AND "X-Linked"[Title/Abstract]) OR (("genes, x linked"[MeSH Terms] OR ("genes"[All Fields] AND "X-Linked"[All Fields]) OR "x-linked genes"[All Fields] OR "X-Linked"[All Fields]) AND "hypergonadotropic ovarian failure"[Title/Abstract]) OR (("genes, x linked"[MeSH Terms] OR ("genes"[All Fields] AND "X-Linked"[All Fields]) OR "x-linked genes"[All Fields] OR "X-Linked"[All Fields]) AND "hypergonadotropic ovarian failure"[Title/Abstract]) OR (("menopause, premature"[MeSH Terms] OR ("menopause"[All Fields] AND "Premature"[All Fields]) OR "premature menopause"[All Fields] OR ("Premature"[All Fields] AND "Ovarian"[All Fields] AND "Failure"[All Fields]) OR "premature ovarian failure"[All Fields]) AND "X-Linked"[Title/Abstract]) OR (("menopause, premature"[MeSH Terms] OR ("menopause"[All Fields] AND "Premature"[All Fields]) OR "premature menopause"[All Fields] OR ("Premature"[All Fields] AND "Ovarian"[All Fields] AND "Failure"[All Fields]) OR "premature ovarian failure"[All Fields]) AND "X-Linked"[Title/Abstract]) OR "fragile x associated primary ovarian insufficiency"[Title/Abstract] OR "fragile x associated primary ovarian insufficiency"[Title/Abstract] OR (("Fragile"[All Fields] AND "X"[All Fields]) AND "premature ovarian failure"[Title/Abstract]) OR "fmr1 related primary ovarian insufficiency"[Title/Abstract] OR "fmr1 related primary ovarian insufficiency"[Title/Abstract] OR "primary ovarian insufficiency fragile x associated"[Title/Abstract] OR "primary ovarian insufficiency fragile x associated"[Title/Abstract]) OR (("respon"[Title/Abstract] OR "ovarian reserve"[Title/Abstract]) AND ("poor"[Title/Abstract] OR "low"[Title/Abstract] OR "slow"[Title/Abstract] OR "inadequate"[Title/Abstract] OR "suboptimal"[Title/Abstract] OR "decreas*"[Title/Abstract] OR "diminish*"[Title/Abstract])) | 146035 |
| #3 | "randomized controlled trial"[Publication Type] OR "controlled clinical trial"[Publication Type] OR "randomized"[Title/Abstract] OR "placebo"[Title/Abstract] OR "clinical trials"[Title/Abstract] OR "randomly"[Title/Abstract] | 1495392 |
| #4 | #1 AND #2 AND #3 | 758 |

**Supplementary Table S2.** Subgroup analyses on clinical pregnancy rate

| **Subgroup** | **No. of studies** | **No. of women** | **Effect Estimate OR (95% CI)** | ***I²*** | ***P*** |
| --- | --- | --- | --- | --- | --- |
| Intervention type | | | | | |
| CoQ10 | 6 | 666 | 2.22 (1.57, 3.14) | 0% | < 0.00001 |
| Melatonin | 8 | 463 | 1.24 (0.75, 2.03) | 0% | 0.40 |
| Myo-inositol | 3 | 184 | 2.61 (0.57, 11.88) | 0% | 0.21 |
| Vitamins | 2 | 737 | 1.11 (0.55, 2.23) | 61% | 0.77 |
| Combined antioxidants | 2 | 168 | 1.45 (0.40, 5.21) | 73% | 0.57 |
| Treatment duration | | | | | |
| During COS | 7 | 362 | 1.73 (1.02, 2.93) | 0% | 0.04 |
| 1 month before the COS | 2 | 192 | 2.07 (0.50, 8.53) | 0% | 0.31 |
| 2 months before the COS | 2 | 225 | 1.62 (0.86, 3.05) | 0% | 0.13 |
| 3 months before the COS | 6 | 600 | 1.94 (1.16, 3.24) | 39% | 0.01 |
| 2 to 12 weeks before the COS | 1 | 630 | 0.86 (0.62, 1.18) | NA | 0.34 |
| Population | | | | | |
| > 35 years old with diminished ovarian reserve | 7 | 520 | 2.13 (1.27, 3.57) | 0% | 0.004 |
| > 35 years old with suboptimal ovarian response | 11 | 1199 | 1.08 (0.84, 1.39) | 0% | 0.54 |
| < 35 years old with diminished ovarian reserve | 3 | 499 | 1.66 (0.74, 3.70) | 75% | 0.22 |

*CI, confidence interval; CoQ10, coenzyme Q10; COS, controlled ovarian stimulation; NA, not available; OR, odds ratio.*

**Supplementary Table S3.** Subgroup analysis on oocytes and embryos outcomes

| **Subgroup** | **No. of studies** | **No. of women** | **Effect Estimate MD (95% CI)** | ***I²*** | ***P*** |
| --- | --- | --- | --- | --- | --- |
| **Number of retrieved oocytes** | | | | | |
| Intervention type | | | | | |
| CoQ10 | 4 | 532 | 1.51 (1.13, 1.89) | 11% | < 0.00001 |
| Melatonin | 3 | 255 | 0.35 (-0.47, 1.16) | 0% | 0.40 |
| Myo-inositol | 2 | 172 | 0.45 (-0.61, 1.51) | 56% | 0.41 |
| Vitamins | 2 | 673 | 0.21 (-1.35, 1.78) | 81% | 0.79 |
| Combined antioxidants | 1 | 90 | 1.60 (1.36, 1.84) | NA | < 0.00001 |
| Treatment duration | | | | | |
| During COS | 4 | 355 | -0.02 (-0.68, 0.63) | 2% | 0.94 |
| One month before the COS | 1 | 122 | 0.00 (-0.76, 0.76) | NA | 1.00 |
| Two months before the COS | 1 | 169 | 2.00 (1.33, 2.67) | NA | < 0.00001 |
| Three months before the COS | 5 | 513 | 1.51 (1.31, 1.72) | 0% | < 0.00001 |
| 2 to 12 weeks before the COS | 1 | 573 | 1.00 (0.09, 1.91) | NA | 0.03 |
| Population | | | | | |
| > 35 years old with diminished ovarian reserve | 4 | 350 | 0.97 (0.29, 1.64) | 68% | 0.005 |
| > 35 years old with suboptimal ovarian response | 6 | 1018 | 0.69 (-0.20, 1.59) | 73% | 0.13 |
| < 35 years old with diminished ovarian reserve | 2 | 354 | 1.81 (1.12, 2.49) | 12% | < 0.00001 |
| **Number of top-quality embryos** | | | | | |
| Intervention type | | | | | |
| CoQ10 | 3 | 347 | 0.62 (0.32, 0.92) | 64% | < 0.0001 |
| Melatonin | 4 | 321 | 0.64 (0.26, 1.02) | 10% | 0.0010 |
| Vitamins | 2 | 673 | -0.14 (-0.32, 0.04) | 13% | 0.14 |
| Treatment duration | | | | | |
| During COS | 4 | 355 | 0.45 (-0.14, 1.04) | 71% | 0.14 |
| 1 month before the COS | 1 | 66 | 0.82 (0.12, 1.52) | NA | 0.02 |
| 2 months before the COS | 1 | 169 | 1.00 (0.58, 1.42) | NA | < 0.00001 |
| 3 months before the COS | 2 | 178 | 0.47 (0.30, 0.64) | 0% | < 0.00001 |
| 2 to 12 weeks before the COS | 1 | 573 | 0.00 (-0.31, 0.31) | NA | 1.00 |
| Population |  |  |  |  |  |
| > 35 years old with diminished ovarian reserve | 3 | 244 | 0.49 (0.32, 0.65) | 0% | < 0.00001 |
| > 35 years old with suboptimal ovarian response | 5 | 928 | 0.24 (-0.15, 0.64) | 66% | 0.23 |
| < 35 years old with diminished ovarian reserve | 1 | 169 | 1.00 (0.58, 1.42) | NA | < 0.00001 |

*CI, confidence interval; CoQ10, Coenzyme Q10; COS, controlled ovarian stimulation; MD, mean difference; NA, not available.*

**Supplementary Table S4.** Subgroup analysis on gonadotropin dose

| **Subgroup** | **No. of studies** | **No. of women** | **Effect Estimate MD (95% CI)** | ***I²*** | ***P*** |
| --- | --- | --- | --- | --- | --- |
| Intervention type | | | | | |
| CoQ10 | 2 | 255 | -752.19 (-1233.18, -271.19) | 48% | 0.002 |
| Melatonin | 1 | 66 | 115.00 (-525.10, 755.10) | NA | 0.72 |
| Myo-inositol | 2 | 124 | -256.16 (-701.29, 188.98) | 0% | 0.26 |
| Vitamins | 2 | 673 | -70.47 (-220.65, 79.71) | 13% | 0.36 |
| Combined antioxidants | 1 | 90 | -212.80 (-269.36, -156.24) | NA | < 0.00001 |
| Treatment duration | | | | | |
| During COS | 1 | 100 | -199.60 (-481.28, 81.08) | NA | 0.16 |
| 1 month before the COS | 2 | 178 | -135.58 (-538.49, 267.33) | 0% | 0.51 |
| 2 months before the COS | 1 | 169 | -1075.00 (-1699.58, -450.42) | NA | 0.0007 |
| 3 months before the COS | 2 | 176 | -344.83 (-679.47, -10.19) | 74% | 0.04 |
| More than one month | 1 | 12 | -133.30 (-1001.29, 734.69) | NA | 0.76 |
| 2 to 12 weeks before the COS | 1 | 573 | -25.00 (-175.56, 125.56) | NA | 0.74 |
| Population | | | | | |
| > 35 years old with diminished ovarian reserve | 4 | 276 | -325.22 (-623.51, -26.94) | 20% | 0.03 |
| > 35 years old with suboptimal ovarian response | 3 | 763 | -148.85 (-283.82, -13.88) | 62% | 0.03 |
| < 35 years old with diminished ovarian reserve | 1 | 169 | -1075.00 (-1699.58, -450.42) | NA | 0.0007 |

*CI, confidence interval; CoQ10, Coenzyme Q10; COS, controlled ovarian stimulation; MD, mean difference; NA, not available.*

**Supplementary Table S5.** Subgroup analysis of melatonin on number of high-quality embryos

| **Subgroup** | **No. of studies** | **No. of women** | **Effect Estimate MD (95% CI)** | ***I²*** | ***P*** |
| --- | --- | --- | --- | --- | --- |
| **Dose** | | | | | |
| 0-5 mg/d | 3 | 135 | 0.99 (0.04, 1.95) | 23% | 0.04 |
| 5-10 mg/d | 3 | 132 | 0.68 (-0.17, 1.53) | 29% | 0.12 |
| > 10 mg/d | 1 | 54 | 0.50 (-1.19, 2.19) | NA | 0.56 |
| **Treatment duration** | | | | | |
| During COS | 6 | 255 | 0.70 (0.09, 1.30) | 18% | 0.02 |
| 1 month before the COS | 1 | 66 | 0.82 (0.12, 1.52) | NA | 0.02 |
| **Population** | | | | | |
| > 35 years old with diminished ovarian reserve | 1 | 66 | 0.82 (0.12, 1.52) | NA | 0.02 |
| > 35 years old with suboptimal ovarian response | 6 | 255 | 0.70 (0.09, 1.30) | 18% | 0.02 |

*CI, confidence interval; COS, controlled ovarian stimulation; MD, mean difference; NA, not available.*

**Supplementary Table S6.** Meta-regression analyses of outcomes and moderator variables

| **Outcomes** | **Coefficient** | **Standard Error** | ***t*** | ***P*** | **95% CI** |
| --- | --- | --- | --- | --- | --- |
| **Clinical pregnancy rate** | | | | | |
| Antioxidant type | -0.123 | 0.095 | -1.29 | 0.217 | -0.325, 0.079 |
| Treatment duration | -0.075 | 0.070 | -1.07 | 0.299 | -0.222, 0.073 |
| Population | -0.070 | 0.197 | -0.36 | 0.726 | -0.488, 0.347 |
| **Number of retrieved oocytes** |  |  |  |  |  |
| Antioxidant type | -0.042 | 0.123 | -0.34 | 0.740 | -0.313, 0.229 |
| Treatment duration | 0.409 | 0.154 | 2.66 | 0.022 | 0.071, 0.748 |
| Population | 0.426 | 0.307 | 1.39 | 0.192 | -0.249, 1.101 |
| **Number of MII oocytes** |  |  |  |  |  |
| Antioxidant type | 0.195 | 0.470 | -0.17 | 0.872 | -1.346, 1.735 |
| Treatment duration | -0.082 | 0.470 | -0.17 | 0.872 | -1.578, 1.414 |
| Population | -0.559 | 1.315 | -0.42 | 0.700 | -4.743, 3.626 |
| **Number of high-quality embryos** | | | | | |
| Antioxidant type | -0.480 | 0.186 | -2.58 | 0.033 | -0.910, -0.051 |
| Treatment duration | 0.150 | 0.127 | 1.18 | 0.272 | -0.143, 0.443 |
| Population | 0.310 | 0.280 | 1.11 | 0.301 | -0.336, 0.955 |
| **Dose of gonadotrophin** |  |  |  |  |  |
| Antioxidant type | 152.943 | 83.519 | 1.83 | 0.141 | -78.944, -384.830 |
| Treatment duration | 29.141 | 68.035 | 0.43 | 0.690 | -159.753, 218.035 |
| Population | -232.870 | 192.913 | -1.21 | 0.294 | -768.482, 302.742 |

*CI, confidence interval; MII, metaphase II.*

**Supplementary Figure S1.** Risk of bias assessment of included studies

A


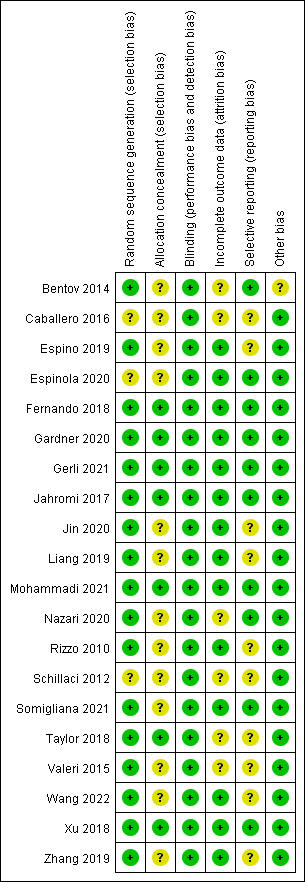

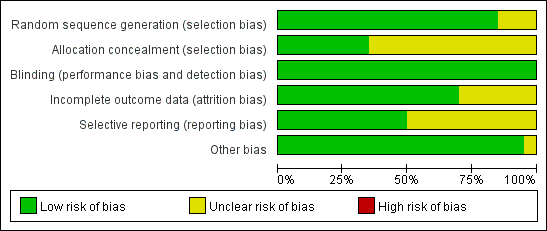
(A) Risk of bias graph. (B) Risk of bias summary.

B
